# Supplementary material for: Pathogenesis-Related 1 (PR1) Protein Family Genes Involved in Sugarcane Responses to Ustilago scitaminea Stress
Source: Int J Mol Sci. 2024 Jun 12;25(12):6463. doi: 10.3390/ijms25126463 (PMC11203535; doi:10.3390/ijms25126463)
Supplement: Supplementary file 1 [file ijms-25-06463-s001.zip › ijms-2998133-supplementary.pdf]

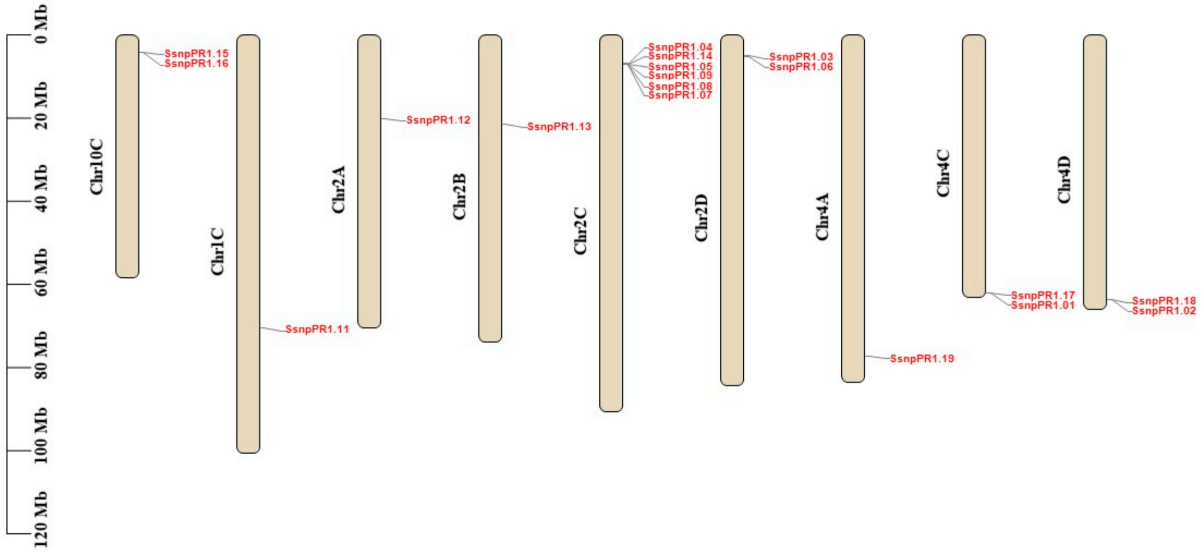

Figure S1: Chromosomal localization of SsnpPR1 genes. Chromosome and gene names are indicated by black and red colors on the outer side of bars, respectively.

Table S1: Detailed information about SsnpPR1 orthologs.

Sr. No.	Query item	String ID	Identity	Bit score	Preferred name	Annotation
1	SpnpPR1.01	3702.AT4G31470.1	42.2	119.4	AT4G31470	Pathogenesis-related protein homolog; CAP (cysteine-rich secretory proteins, antigen 5, and pathogenesis-related 1 protein) superfamily protein. Its function is described as molecular_function unknown. Involved in biological_process unknown. Located in endomembrane system, extracellular region. Expressed in root. Contains the following InterPro domains: Allergen V5/Tpx-1 related, conserved site (InterPro:IPR018244), Allergen V5/Tpx-1 related (InterPro:IPR001283), Ves allergen (InterPro:IPR002413), SCP-like extracellular (InterPro:IPR014044). BEST Arabidopsis thaliana protein match is: [...]
2	SpnpPR1.02	3702.AT4G31470.1	42.2	119.4	AT4G31470	Pathogenesis-related protein homolog; CAP (cysteine-rich secretory proteins, antigen 5, and pathogenesis-related 1 protein) superfamily protein. Its function is described as molecular_function unknown. Involved in biological_process unknown. Located in endomembrane system, extracellular region. Expressed in root. Contains the following InterPro domains: Allergen V5/Tpx-1 related, conserved site (InterPro:IPR018244), Allergen V5/Tpx-1 related (InterPro:IPR001283), Ves allergen (InterPro:IPR002413), SCP-like extracellular (InterPro:IPR014044). BEST Arabidopsis thaliana protein match is: [...]
3	SpnpPR1.03	3702.AT5G57625.1	54.7	170.2	AT5G57625	CAP (cysteine-rich secretory proteins, antigen 5, and pathogenesis-related 1 protein) superfamily protein. Its function is described as molecular_function unknown. Involved in biological_process unknown. Located in endomembrane system, extracellular region. Contains the following InterPro domains: Allergen V5/Tpx-1 related,

						conserved site (InterPro:IPR018244), Allergen V5/Tpx-1 related (InterPro:IPR001283), Ves allergen (InterPro:IPR002413), SCP-like extracellular (InterPro:IPR014044). BEST Arabidopsis thaliana protein match is: CAP (cysteine-rich secretory proteins, antigen 5, and pat [...]
4	SpnpPR1.04	3702.AT 5G57625 .1	57.5	179.9	AT5G57625	CAP (cysteine-rich secretory proteins, antigen 5, and pathogenesis-related 1 protein) superfamily protein. Its function is described as molecular_function unknown. Involved in biological_process unknown. Located in endomembrane system, extracellular region. Contains the following InterPro domains: Allergen V5/Tpx-1 related, conserved site (InterPro:IPR018244), Allergen V5/Tpx-1 related (InterPro:IPR001283), Ves allergen (InterPro:IPR002413), SCP-like extracellular (InterPro:IPR014044). BEST Arabidopsis thaliana protein match is: CAP (Cysteine-rich secretory proteins, Antigen 5, and Pat [...]
5	SpnpPR1.05	3702.AT 4G25790 .1	46	145.2	AT4G25790	CAP (cysteine-rich secretory proteins, antigen 5, and pathogenesis-related 1 protein) superfamily protein. Its function is described as molecular_function unknown; Involved in biological_process unknown. Located in endomembrane system, extracellular region. Expressed in root. Contains the following InterPro domains: Allergen V5/Tpx-1 related, conserved site (InterPro:IPR018244), Allergen V5/Tpx-1 related (InterPro:IPR001283), Ves allergen (InterPro:IPR002413), SCP-like extracellular (InterPro:IPR014044). BEST Arabidopsis thaliana protein match is: CAP (cysteine-rich secretory proteins, [...]

6	SpnpPR1.06	3702.AT4G33720.1	47.5	147.5	AT4G33720	Putative pathogenesis-related protein 1, 19.3K; CAP (cysteine-rich secretory proteins, antigen 5, and pathogenesis-related 1 protein) superfamily protein. Its function is described as molecular_function unknown. Involved in biological_process unknown. Located in endomembrane system, extracellular region. Expressed in root. Contains the following InterPro domains: Allergen V5/Tpx-1 related, conserved site (InterPro:IPR018244), Allergen V5/Tpx-1 related (InterPro:IPR001283), Ves allergen (InterPro:IPR002413), SCP-like extracellular (InterPro:IPR014044). BEST Arabidopsis thaliana protein [...]
7	SpnpPR1.07	3702.AT4G33720.1	46.2	144.4	AT4G33720	Putative pathogenesis-related protein 1, 19.3K; CAP (cysteine-rich secretory proteins, antigen 5, and pathogenesis-related 1 protein) superfamily protein. Its function is described as molecular_function unknown. Involved in biological_process unknown. Located in endomembrane system, extracellular region. Expressed in root. Contains the following InterPro domains: Allergen V5/Tpx-1 related, conserved site (InterPro:IPR018244), Allergen V5/Tpx-1 related (InterPro:IPR001283), Ves allergen (InterPro:IPR002413), SCP-like extracellular (InterPro:IPR014044). BEST Arabidopsis thaliana protein [...]
8	SpnpPR1.08	3702.AT3G09590.1	43.4	146.7	AT3G09590	CAP (cysteine-rich secretory proteins, antigen 5, and pathogenesis-related 1 protein) superfamily protein. Its function is described as molecular_function unknown. Involved in biological_process unknown. Located in endomembrane system, extracellular region. Expressed in male gametophyte, pollen tube. Expressed during L mature pollen stage, M germinated pollen stage. Contains

						the following InterPro domains: Allergen V5/Tpx-1 related, conserved site (InterPro:IPR018244), Allergen V5/Tpx-1 related (InterPro:IPR001283), Ves allergen (InterPro:IPR002413), SCP-like extracellular (InterPro:IP [...]
9	SpnpPR1.09	3702.AT 4G33720 .1	46.3	126.7	AT4G33720	Putative pathogenesis-related protein 1, 19.3K; CAP (cysteine-rich secretory proteins, antigen 5, and pathogenesis-related 1 protein) superfamily protein. Its function is described as molecular_function unknown. Involved in biological_process unknown. Located in endomembrane system, extracellular region. Expressed in root. Contains the following InterPro domains: Allergen V5/Tpx-1 related, conserved site (InterPro:IPR018244), Allergen V5/Tpx-1 related (InterPro:IPR001283), Ves allergen (InterPro:IPR002413), SCP-like extracellular (InterPro:IPR014044). BEST Arabidopsis thaliana protein [...]
10	SpnpPR1.11	3702.AT 3G09590 .1	51.5	172.6	AT3G09590	CAP (cysteine-rich secretory proteins, antigen 5, and pathogenesis-related 1 protein) superfamily protein. Its function is described as molecular_function unknown. Involved in biological_process unknown. Located in endomembrane system, extracellular region. Expressed in male gametophyte, pollen tube. Expressed during L mature pollen stage, M germinated pollen stage. Contains the following InterPro domains: Allergen V5/Tpx-1 related, conserved site (InterPro:IPR018244), Allergen V5/Tpx-1 related (InterPro:IPR001283), Ves allergen (InterPro:IPR002413), SCP-like extracellular (InterPro:IP [...]
11	SpnpPR1.12	3702.AT 2G14610 .1	51.2	162.9	PR1	Pathogenesis-related protein 1. PR1 gene expression is induced in response to a variety of pathogens. It is a useful molecular marker for the SAR response. Though

						the Genbank record for the cDNA associated with this gene is called ‘PR-1-like’, the sequence actually corresponds to PR1. Expression of this gene is salicylic acid responsive.
12	SpnpPR1.13	3702.AT 5G57625 .1	56.6	165.6	AT5G57625	CAP (cysteine-rich secretory proteins, antigen 5, and pathogenesis-related 1 protein) superfamily protein. Its function is described as molecular_function unknown. Involved in biological_process unknown. Located in endomembrane system, extracellular region. Contains the following InterPro domains: Allergen V5/Tpx-1 related, conserved site (InterPro:IPR018244), Allergen V5/Tpx-1 related (InterPro:IPR001283), Ves allergen (InterPro:IPR002413), SCP-like extracellular (InterPro:IPR014044). BEST Arabidopsis thaliana protein match is: CAP (cysteine-rich secretory proteins, antigen 5, and pat [...]
13	SpnpPR1.14	3702.AT 5G57625 .1	59	186	AT5G57625	CAP (cysteine-rich secretory proteins, antigen 5, and pathogenesis-related 1 protein) superfamily protein. Its function is described as molecular_function unknown. Involved in biological_process unknown. Located in endomembrane system, extracellular region. Contains the following InterPro domains: Allergen V5/Tpx-1 related, conserved site (InterPro:IPR018244), Allergen V5/Tpx-1 related (InterPro:IPR001283), Ves allergen (InterPro:IPR002413), SCP-like extracellular (InterPro:IPR014044). BEST Arabidopsis thaliana protein match is: CAP (cysteine-rich secretory proteins, antigen 5, and pat [...]
14	SpnpPR1.15	3702.AT 2G14610 .1	56.3	179.5	PR1	Pathogenesis-related protein 1. PR1 gene expression is induced in response to a variety of pathogens. It is a useful molecular marker for the SAR response. Though the Genbank record for the cDNA associated with this gene is called ‘PR-1-like’, the sequence actually

						corresponds to PR1. Expression of this gene is salicylic acid responsive.
15	SpnpPR1.16	3702.AT 2G14610 .1	56.3	179.5	PR1	Pathogenesis-related protein 1. PR1 gene expression is induced in response to a variety of pathogens. It is a useful molecular marker for the SAR response. Though the Genbank record for the cDNA associated with this gene is called 'PR-1-like', the sequence actually corresponds to PR1. Expression of this gene is salicylic acid responsive.
16	SpnpPR1.17	3702.AT 5G57625 .1	52.1	151.4	AT5G57625	CAP (cysteine-rich secretory proteins, antigen 5, and pathogenesis-related 1 protein) superfamily protein. Its function is described as molecular_function unknown. Involved in biological_process unknown. Located in endomembrane system, extracellular region. Contains the following InterPro domains: Allergen V5/Tpx-1 related, conserved site (InterPro:IPR018244), Allergen V5/Tpx-1 related (InterPro:IPR001283), Ves allergen (InterPro:IPR002413), SCP-like extracellular (InterPro:IPR014044). BEST Arabidopsis thaliana protein match is: CAP (cysteine-rich secretory proteins, antigen 5, and pat [...]
17	SpnpPR1.18	3702.AT 5G57625 .1	52.1	151.4	AT5G57625	CAP (cysteine-rich secretory proteins, antigen 5, and pathogenesis-related 1 protein) superfamily protein. Its function is described as molecular_function unknown. Involved in biological_process unknown. Located in endomembrane system, extracellular region. Contains the following InterPro domains: Allergen V5/Tpx-1 related, conserved site (InterPro:IPR018244), Allergen V5/Tpx-1 related (InterPro:IPR001283), Ves allergen (InterPro:IPR002413), SCP-like extracellular (InterPro:IPR014044). BEST Arabidopsis thaliana protein match is: CAP (cysteine-rich secretory proteins, antigen 5, and pat [...]

18	SnpPR1.19	3702.AT 5G57625 .1	52.1	151	AT5G57625	CAP (cysteine-rich secretory proteins, antigen 5, and pathogenesis-related 1 protein) superfamily protein. Its function is described as molecular_function unknown. Involved in biological_process unknown. Located in endomembrane system, extracellular region. Contains the following InterPro domains: Allergen V5/Tpx-1 related, conserved site (InterPro:IPR018244), Allergen V5/Tpx-1 related (InterPro:IPR001283), Ves allergen (InterPro:IPR002413), SCP-like extracellular (InterPro:IPR014044). BEST Arabidopsis thaliana protein match is: CAP (cysteine-rich secretory proteins, antigen 5, and pat [...]
